# On Gouty Dyspepsia, or Gout of the Stomach

**Published:** 1881-05

**Authors:** Germain Sée


### Original Translations.

ARTICLE X.

### ON GOUTY DYSPEPSIA, OR GOUT OF THE STOMACH. By Prof. Germain Sée.

Gouty dyspepsia, or gastric gout, is surely a rarer affection than is generally asserted; its existence is altogether denied by Watson, who refers it to common violent irritations following excesses in eating. Brinton asks, what is understood by gout of the stomach, and comes to the conclusion: There may be hepatic colics, or uræmic manifestations, which in gouty subjects are said to be a metastasis of gout to the stomach, but, besides these errors, there is nothing left constituting a special disease, nothing worthy the denomination of gastric gout, a term which patients, imbued with medical principles, abuse in so strange a manner; but this negation, this willful disregard of facts, adds more evidence to the truth that this is an obscure subject, and these symptoms which cannot be denied, are not easily accounted for.

My experience with these various forms of gout has demonstrated to me the following:

(*a*.) There exists an *ante-gouty*, *pseudo-dyspepsia;* it is a forerunner of gout, a first premonitory symptom, coming on very early so that Ford said it was the cause of that disease. Patients complain of digestive disturbances, with feelings of compression, squeezing, or more generally of fullness in the epigastrium, of distension of the abdomen, eructation, pyrosis, tympanitis, constipation, and hæmorrhoids; this affection has been described of old, and is yet known in Germany under the name of *abdominal venous plethora.* Is that a true dyspepsia? Not at all. It is one of those atonic states of the intestine, with or without constipation, with or without hæmorrhoids, which are often observed to follow excesses in eating, in atonic, weak or obese individuals; it is a false dyspepsia, deserving the title *ante-gouty* in the sense that it is the *habitual temperament* of gouty subjects.

(*b.*) *Acute paroxysmal catarrhal dyspepsia.* Before the crises of gout, and while they last, we often observe signs of the condition commonly known as gastric troubles, or acute mucous catarrh. The patient loses appetite, his tongue becomes coated, his digestion slow, difficult, with metorism and eructations, is always accompanied by much uneasiness, by a sensation of lassitude and despondency, as if a serious disease was impending. There are patients who never suffer crises of gout, but these are preceded by such gastric trouble, as a sort of premonitory symptom unaccompanied with fever. This gastric difficulty may last through the whole attack, especially if it is a severe one. Is that a truly gouty phenomenon, or is it not one of those perturbations of the secretions, which we might call acute mucous dyspepsia, of the common kind, as we sometimes observe in the beginning of acute diseases? If that state persists during the evolution of articular gout, it ordinarily manifests itself under the form of a persisting anorexia of which pain in the joints is a frequent concomitant. Let us call that state *acute catarrhal dyspepsia.*

(*c.*) *Acute and true urœmic dyspepsia.* Here is presently a series of phenomena which are briskly manifested during an attack of gout, or while this is on the wane; it violently appears as a *cardialgia.* The patient is taken with cramps and colics in the stomach, with constant hiccough and pyrosis, followed by repeated emeses of acrid matter; resembling cases of poisoning, with symptoms pertaining to peritonitis, which may induce lipothymia, or even syncope with cooling of the surface of the body, passive algidity of limbs and face, with a livid discoloration of the lips. Is not this uric acid poisoning? Had injections of that acid into the blood been followed by other than negative results, one would be tempted to ascribe a toxic cause to that cardialgia, referring it to the sudden introduction of an excess of uric acid in the blood; lacking such data, we confine ourselves to its mode of elimination ; for we know that the stomach is the true excretory organ by which urea is thrown out after accumulating in the blood, on account of some alteration of the kidneys; the same is true of uric acid, which is equally eliminated by the stomach ; but the presence of an excretory product in the gastro-intestinal mucous membrane, could not fail chemically to alter the gastric juice and give rise to a serious dyspepsia with all the symptoms of uræmic poisoning. The main point is to find out the cause of this sudden invasion of uric acid into the blood, and thence into the stomach. Here we come to the doctrine of metastasis. If, in reality, cardialgia arises when the crisis in the joint recedes, we may believe that uric acid which had invaded the joints is entirely reabsorbed into the blood, which eliminates it through the stomach; but such is not always the case, this mutual relation is not all constant; cardialgia may take place without any improvement in the joints ; retrocession could not be advocated in cases of that nature ; but it remains just as evident that uric acid, circulating in moderate quantity in the blood, may be eliminated by the mucous membrane of the stomach, giving rise to a series of serious symptoms by its passage.

Why such a sudden elimination? The only solution is that in a certain number of cases of poisoning, as by anthocthonous poisons, there is always some tendency for the elimination to select an excretory organ in particular and leave the ordinary way of excretion. In a clinical point of view, it is now proven that there exist dyspepsias, accompanied with *uræmic cardialgia*, which often bring a real danger to the constitution.

These are the three types of dyspepsia which arise more or less directly from gout, other types are allied to the common affection; a gouty patient may suffer from a dyspepsia depending on an irregular diet, he may be a subject to chronic gastritis (Ebstein); but these are referred to dyspepsias in general, and are devoid of characteristics.—*Le Médecin Praticien*, March 5.

